# One proboscis, two tasks: Adaptations to blood-feeding and nectar-extracting in long-proboscid horse flies (Tabanidae, *Philoliche*)

**DOI:** 10.1016/j.asd.2014.07.003

**Published:** 2014-09

**Authors:** Florian Karolyi, Jonathan F. Colville, Stephan Handschuh, Brian D. Metscher, Harald W. Krenn

**Affiliations:** a Department of Integrative Zoology, University of Vienna, Faculty of Life Science, Althanstrasse 14, 1090 Vienna, Austria; b South African National Biodiversity Institute, Kirstenbosch Research Centre, Private Bag X7, Claremont, Cape Town, South Africa; c VetCore Facility for Research, University of Veterinary Medicine, Vienna, Austria; d Department of Theoretical Biology, University of Vienna, Faculty of Life Science, Althanstrasse 14, 1090 Vienna, Austria

**Keywords:** Diptera, *Philoliche*, Functional morphology, Blood sucking, Nectar-feeding, Mouthparts

## Abstract

Female Pangoniinae in the tabanid fly genus *Philoliche* can display remarkably elongated proboscis lengths, which are adapted for both blood- and nectar-feeding. Apart from their role as blood-sucking pests, they represent important pollinators of the South African flora. This study examines the morphology of the feeding apparatus of two species of long-proboscid Tabanidae: *Philoliche rostrata* and *Philoliche gulosa* – both species display adaptations for feeding from a diverse guild of long-tubed flowers, and on vertebrate blood. The heavily sclerotised proboscis can be divided into two functional units. The short, proximal piercing part is composed of the labrum-epipharynx unit, the hypopharynx and paired mandible and maxilla. The foldable distal part is composed of the prementum of the labium which solely forms the food canal and is responsible for nectar uptake via the apical labella. The proboscis works as a drinking straw, relying on a pressure gradient provided by a two-part suction pump in the head. Both proboscis and body lengths and suction pump dimensions show a significantly correlated allometric relationship with each other. This study provides detailed insights into the adaptations for a dual diet using an elongated sucking proboscis, and considers these adaptations in the context of the evolution of nectar feeding in Brachycera.

## Highlights

• The proboscis of female long-proboscid *Philoliche* consists of two functional units.
• This proximal region composes the piercing, blood sucking part of the mouthparts.
• Only the distal proboscis is adapted to feeding nectar from long-spurred flowers.
• Body and proboscis length show a significant positive relationship in *Philoliche rostrata*.
• Suction pump size and proboscis length show a significant positive relationship.

## 1 Introduction

Long, tubular mouthparts have evolved multiple times convergently in various nectar and blood-feeding insect taxa (Krenn et al., 2005, Krenn and Aspöck, 2012). Within Diptera, these elongated proboscides display characteristic morphological and functional adaptations for feeding either on floral nectar (e.g., in Bombyliidae, Syrphidae and Nemestrinidae) or vertebrate blood (e.g. Culicidae, Simuliidae, Tabanidae and Glossinidae). Proboscides for nectar-feeding are generally characterised by a soft tip region for nectar uptake into the food canal (Krenn et al., 2005). In contrast, most blood-feeding insects use a proboscis characterised by piercing structures, including the paired, elongated laciniae of the maxillae and mandible blades, as well as the labrum and the hypopharynx containing the salivary duct (Chaudonneret, 1990, Krenn and Aspöck, 2012). The food canal within the labrum is separated from the salivary canal and both are usually encompassed by the weaker sclerotised labium. In particularly long-proboscid fly taxa, the greatly elongated part of the proboscis consists of the extended prementum of the labium, bearing the apical labella (Karolyi et al., 2012).

Blood-feeding evolved independently among various taxa of Diptera (Krenn and Aspöck, 2012). In the case of Tabanidae, female flies are generally large-bodied blood feeders, which attack various vertebrates, including cattle and man, using a piercing proboscis.

Long-proboscid Tabanidae appear to have co-evolved with flowering plants since the Late Jurassic (Ren, 1998) and they are considered among the first pollinators of early angiosperms (Labandeira, 1998, Labandeira, 2010). In addition, Tabanidae are the only group of Brachycera combining particular morphological adaptations to nectar uptake and blood-feeding in one suction organ. Female representatives of the Pangoniinae are known to use the elongated prementum for nectar feeding, while the shorter piercing apparatus is responsible for penetrating vertebrate skin and blood uptake (Goodier, 1962, Dierl, 1968, Morita, 2008, Morita, 2011).

Despite their blood-sucking behaviour, long-proboscid Tabanidae from the genus *Philoliche*, together with various genera of Nemestrinidae, are important flower visitors and are considered as keystone species in floristically rich regions of South Africa, e.g. the globally renowned Cape Floristic Region (Goldblatt and Manning, 2000a). Linked into a broad network of co-evolution, they are responsible for pollinating around 170 plant species from various families within well-defined flower guilds (Johnson and Steiner, 1997, Goldblatt and Manning, 2000b, Johnson, 2010). These extraordinarily long-proboscid flies are involved in pollinator-mediated speciation of flowering plants and exhibit proboscis length variations due to local adaptations to geographically varying flower guilds (Goldblatt et al., 1995, Johnson and Steiner, 1997). Within these guilds, plants share a similar flower morphology including an elongated and narrow, straight or slightly curved floral tube usually containing nectar (Goldblatt and Manning, 2000b).

As seen in long-proboscid nemestrinid flies (Karolyi et al., 2012), the proboscis of *Philoliche* can be up to three times as long as the body and is specialised to extract nectar from long-spurred flowers (Morita, 2008). In contrast to strictly nectarivorous Nemestrinidae, female *Philoliche* are anautogenous and require blood for nourishing their developing eggs (Lehane, 2005). In addition, nectar represents an important source of energy for both male and female flies and is necessary to sustain daily activities for both sexes (Downes, 1958, Johnson and Johnson, 1993). Compared to nectar, vertebrate blood is a heterogeneous suspension and its viscosity varies with the diameter of the food tube (Kingsolver and Daniel, 1979, Kim et al., 2011b). Consequently, the feeding apparatus is expected to be different in insects with different feeding habits. Therefore, such a twofold diet should impose particular adaptations on the feeding apparatus in *Philoliche*.

The elongated proboscis of Tabanidae offers a unique opportunity to investigate a proboscis that is adapted to perform a dual task: blood-sucking and nectar-feeding within the same individual. Generally, in nectarivorous insects, the suction pump in the head creates a pressure gradient along the proboscis that enables them to rapidly suck-up nectar from flowers (Eberhard and Krenn, 2005, Davis and Hildebrand, 2006, Borrell and Krenn, 2006, Kim et al., 2011a). Recent studies describing the proboscis of nectar feeding in long-proboscid Lepidoptera (Monaenkova et al., 2012) and Nemestrinidae (Karolyi et al., 2013) indicate that the proboscis acts as a combination of a nanosponge with a drinking straw. However, whether the mouthparts of long-proboscid Tabanidae operate in a similar manner to other long-proboscid insects during nectar feeding remains to be investigated. Additionally, it is unclear if adaptations for blood-feeding place constraints on the requirements for additional adaptations of the feeding apparatus with respect to nectar feeding. Recent studies have shown the importance of allometric relationships in forming elongated mouthpart structures with concomitant increases in suction pump dimensions with increasing proboscid lengths (Karolyi et al., 2013).

Although, studies about the feeding behaviour of long-proboscid Tabanidae provide basic insights into mouthpart morphology (Tetley, 1917, Dierl, 1968, Morita, 2007), detailed examinations of long-proboscid mouthpart morphology are essential for gaining insights into the coevolutionary dynamics of elongated plant corolla lengths and fly proboscis lengths. Therefore, the aim of this study was to examine a feeding apparatus that combines blood and nectar-feeding within a single species. This comprises the functional morphology of the mouthparts, including adaptations of different parts of the proboscis associated with different food sources, the associated musculature responsible for proboscis movement and the suction pump in the head. The study further investigates the allometric relationship between body and proboscis length, and the relationship between suction pump dimensions and proboscis lengths to determine the effects of attaining an elongated proboscis on associated mouthpart structures. Comparisons with other flower-visiting and blood-sucking Diptera are discussed to gain a deeper insight into the evolution of form and function of long-proboscides in brachyceran flies.

## 2 Material & methods

### 2.1 Study species

Several specimens of female *Philoliche rostrata* (Linnaeus, 1764) and *Philoliche gulosa* (Wiedemann, 1828) were collected from various locations from winter-rainfall Western Cape Province and Namaqualand, Northern Cape Province in South Africa: Kamieskroon (*P*. *rostrata*: 30°44′11″S, 18°06′57″E; *N* = 15; *P*. *gulosa*, *N* = 10), Kommetjie (*P*. *rostrata*: 34°14′26″S, 18°32′91″E; *N* = 4) and University of Cape Town (*P*. *rostrata*: 33°95′45″S, 18°46′19″E; *N* = 3). Measurements of body, proboscis and labrum were taken using a digital caliper (Helios Digi-Met 1220; 0.01 mm; Preisser Messtechnik GmbH, Gammertingen, Germany); labella measurements were taken with a micrometre using a stereo microscope.

### 2.2 Micro-CT and serial semithin-sections (SEM)

The internal morphology of the head capsule and proboscis was examined using serial semithin cross section technique and micro-CT.

For serial semithin cross sections, proximal and distal parts of the proboscis were dehydrated to 100% ethanol and subsequently 100% acetone (three times for 15 min each). Specimens were transferred into a 1:1 mixture of pure acetone and Agar Low Viscosity Resin (resin 19.2 g, VH1 hardener 3.2 g, VH2 hardener 17.6 g, LV accelerator 1.0 g; Agar Scientific Ltd., Stansted, UK) for 30 min, followed by a mixture of acetone and resin (3:7) for 16 h and two steps (8 h and 16 h respectively) of 100% Agar Low Viscosity Resin. Dehydration and embedding were conducted at room temperature on a shaker platform. Proboscis parts were embedded in molds with pure Agar Low Viscosity Resin, and finally evacuated three times (100–150 Torr pressure at 40 °C) to ensure a complete infiltration of resin into the specimen. The polymerisation was proceeded at 70 °C within 24 h. Sections of 1 μm were cut with a Leica EM UC6 microtome (Leica Microsystems, Wetzlar, Germany) equipped with a Histo-Jumbo diamond knife (Diatome AG, Biel; Switzerland). Serial sections were transferred onto microscopic slides and stained with a mixture of azure II and methylene blue in hydrous borax solution (1%) diluted 1:9, at 40 °C for 20 s. Microphotographs were taken using a Nikon Eclipse E800 microscope (Nikon, Tokyo, Japan) equipped with a Nikon digital Sight DS-Fi2 camera. Dimensions of the food canal were measured with NIS-Elements Imaging software 4.0.

For micro-CT examination of the head muscles, specimen of *P. rostrata* (*n* = 10) were fixed in 95% ethanol. All specimens were dehydrated to absolute ethanol and stained with 1% iodine in 100% ethanol overnight. Prior to scanning, samples were washed in 100% ethanol (Metscher, 2009). Specimens were scanned with an Xradia MicroXCT-200 system (Carl Zeiss X-ray Microscopy, Inc., Pleasanton, CA; optical lens 2×; tungsten source at 40 kV and 200 μA; reconstructed isotropic voxel size 9.9 μm). For 3D reconstructions of the suction pump, AMIRA (version 5.3.3, FEI Visualization Science Group, Mérignac, France) was used. Suction pump muscles were manually segmented in the AMIRA segmentation editor. Finally, structures were visualised in the AMIRA Viewer using both volume and surface renderings.

### 2.3 Scanning electron microscopy

All micrographs were taken with a Philips XL 20 SEM (Philips, Amsterdam, Netherlands) using the standard procedures for scanning electron microscopy (Bock, 1987). Proboscides were dehydrated in 100% ethanol and submerged in Hexamethyldisilazan. After air drying overnight, specimens were mounted on viewing stubs with graphite adhesive tape and sputter-coated with gold (300 s, Agar sputtercoater B7340).

### 2.4 Statistics

Data analyses were conducted using the statistic software R 2.15.2 (R Core Team, 2012). Since the data were not normally distributed proboscis length and body length were correlated using permutation tests for linear models within the ‘lmPerm package’ (Wheeler, 2010). Correlations between labrum and labella length with the overall proboscis length were calculated using the ‘Hmisc package’ (Harrell Jr, 2013).

Furthermore, relations between proboscis length and suction pump dimensions were also calculated using the ‘lmPerm package’ (Wheeler, 2010) for cibarial dilator muscles, pharyngeal dilator and compressor muscles, as well as for cibarial retractor and protractor muscles.

## 3 Results

### 3.1 Mouthpart morphology

**Fig. 1:**
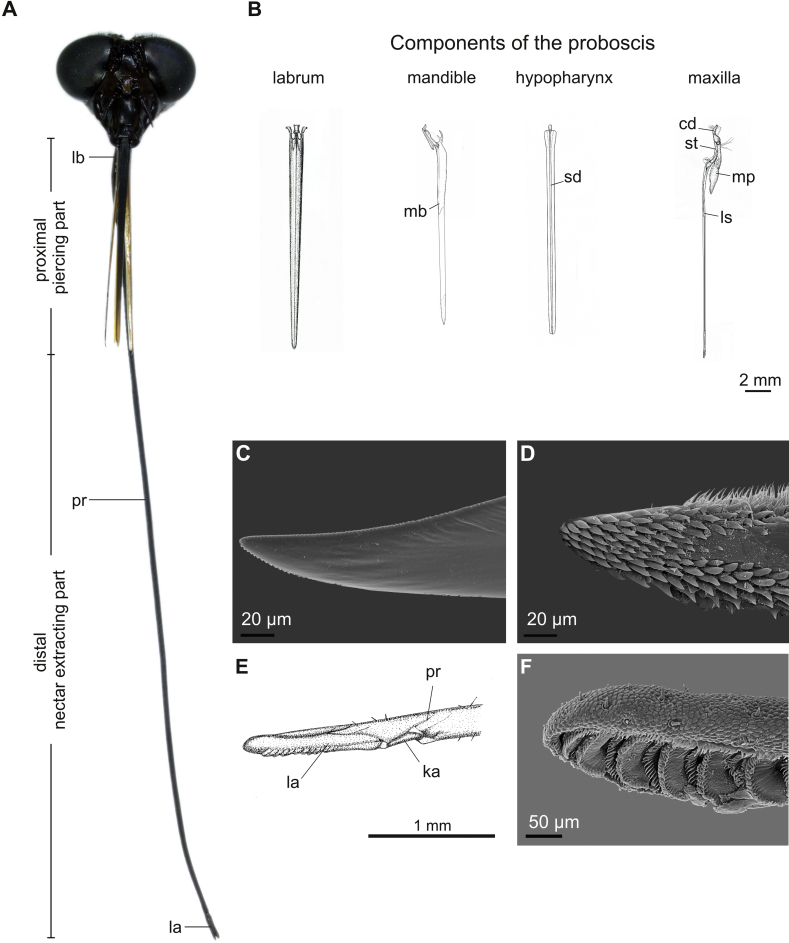
Head and mouthparts of long-proboscid horse fly *Philoliche rostrata*. **A**. Head with extended proboscis divided into the proximal piercing part and the distal part responsible for nectar intake, including the apical labella (la). The prementum (pr) continues from the membranous labial base (lb) and reaches over the entire length of the proboscis. **B**. Dorsal view of biting structures; labrum, mandible blade (mb), hypopharynx with salivary duct (sd) and maxilla. Each maxilla consists of a basal cardo (cd) and stipes (st) carrying the two segmented maxillary palp (mp) and the lacinia stylet (ls). **C**–**D**. SEM micrographs of serrated mandible tip (C) and toothed lacinia tip (D). **E**. Apical prementum with labella joint via the kappa (ka). **F**. SEM micrographs of opened labella displaying the pseudotracheal system.

The proboscis of female *Philoliche* was divided into two functional units (Fig. 1A). (1) The short proximal piercing part included the labrum-epipharynx unit, paired mandibles, paired maxillary structures and the hypopharynx. This proximal region composed the piercing and blood sucking part of the mouthparts. (2) The labium ranged over the whole proboscis length, and the prementum alone formed the extremely elongated distal part that was responsible for nectar uptake via the short apical labella.

**Fig. 2:**
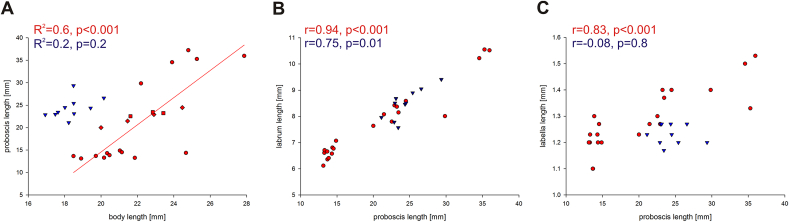
Allometric relationships in the proboscis of *Philoliche rostrata* (red symbols) and *P*. *gulosa* (blue symbols). **A**. Linear regressions between the body and proboscis length of *P*. *rostrata* and *P*. *gulosa*. The different study sites are displayed with different symbols. Study sites: Kamieskroon (circles), Kommetjie (diamonds) and University of Cape Town (squares).**B**. Correlation between overall proboscis length and labrum length. **C**. Correlation between overall proboscis length and labella length. (For interpretation of the references to colour in this figure legend, the reader is referred to the web version of this article.)

The biometry of the proboscis is summarised in Table 1. The proboscis of both *Philoliche* species was up to twofold longer than the body. Linear regressions revealed a significant positive relationship between body and overall proboscis length for *P*. *rostrata*, but not for *P*. *gulosa* (Fig. 2A). However, in both species correlations revealed a significant positive relationship between prementum and labrum length (Fig. 2B). Correlations between labella and proboscis length revealed a positive relationship only in *P*. *rostrata* (Fig. 2C).

**Table 1:** Proboscis measurements of *Philoliche* sp.

	*P. rostrata* (*n* = 22)		*P. gulosa* (*n* = 10)	
	Length [mm]	Mean ± s.d.	Length [mm]	Mean ± s.d.
Labrum	6–11	7.86 ± 4.45	7–9	8.84 ± 0.58
Prementum	13–37	21.36 ± 8.41	21–29	24.37 ± 2.31
Labella	1–1.5	1.29 ± 0.11	1.2–1.3	1.23 ± 0.04

In *P*. *rostrata*, the proximal part, measured as the length of the labrum, displayed a high length variation compared to *P*. *gulosa*. In contrast, labella length showed a low variation in length in both species (Table 1).

#### 3.1.1 Proximal piercing part

**Fig. 3:**
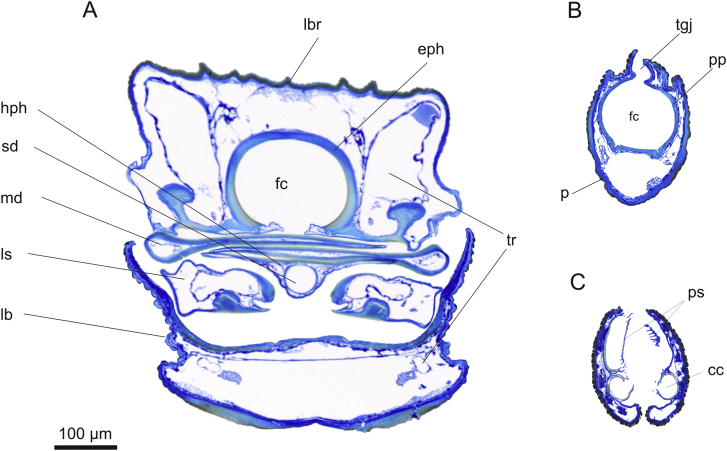
Proboscis cross sections of *Philoliche rostrata*. **A**. Proximal part; labrum (lbr) with epipharyngeal (eph) food canal (fc), mandibles (md) and laciniae stylet (ls), in between hypopharynx (hph) with salivary duct (sd). The piercing structures are sheathed in the labium (lb). **B**. Distal part; prementum (p) containing the labial food canal (fc) lined by the paraphyses (pp) and closed by a dorsal tongue and groove joint (tgj). **C**. Apical labella with pseudotracheal system (ps) and collecting canals (cc).

The strongly sclerotised labrum measured about one third of the proboscis and formed the dorsal cover of the proximal food canal (Fig. 1A, B). At the base, it was articulated with the clypeus via three small sclerites and the dorsal rostral membrane. In cross section, the labrum contained an omega-shaped half pipe lined with the sclerotised epipharynx which formed the nutrition canal (Fig. 3A). The epipharynx was connected with the dorsal cibarial wall, joining the food canal with the suction pump within the head capsule. The labrum was laterally stiffened by cuticular ledges and traversed by large tracheae on each side.

**Fig. 4:**
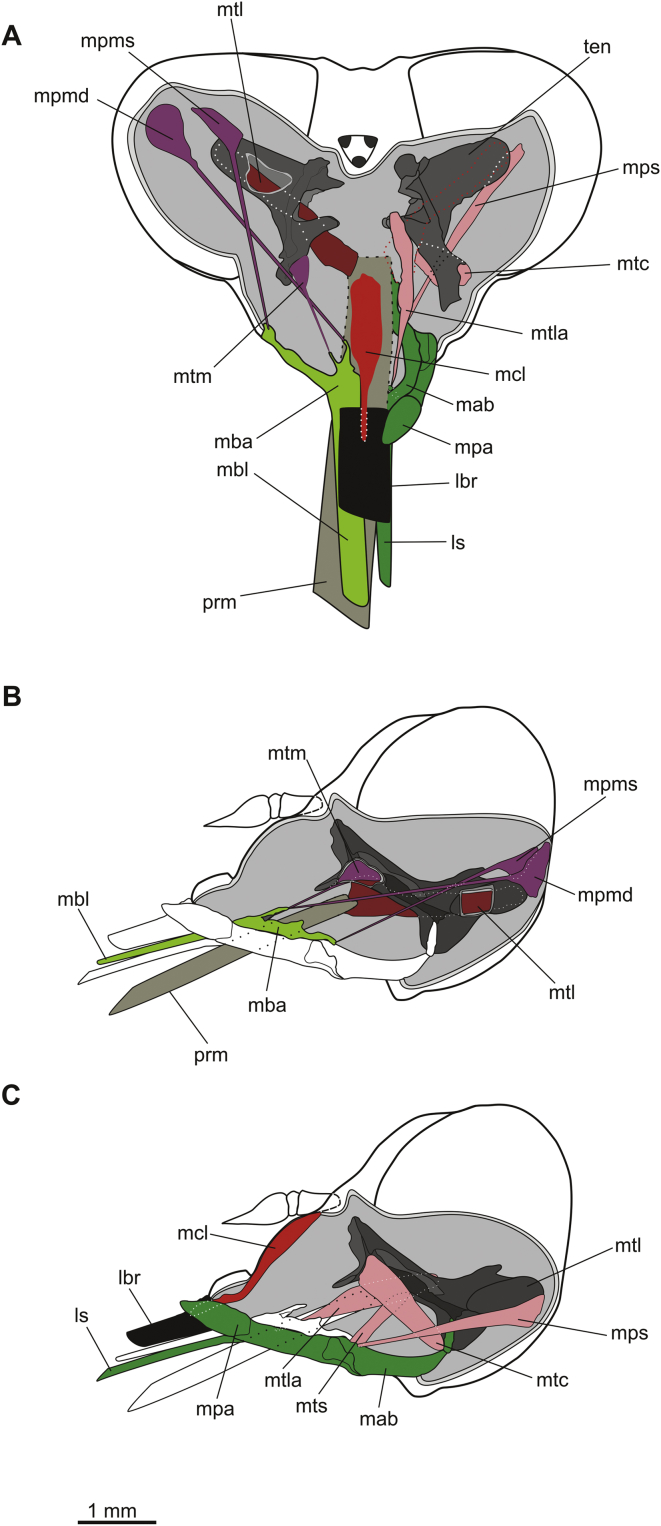
Schematic illustration of the head and cut proboscis with mouthpart musculature of *Philoliche rostrata*. **A**. Frontal view with left mandible, right maxilla, labrum and prementum with the attached musculature: mandible muscles (purple), maxilla musculature (pink), labrum muscle (bright red). The tentorium arm on the left is additionally cut open to display a part of the labium muscle (dark red). **B**. Lateral view with mandible and prementum muscles. The tentorium is partly cut to display the tentorial adductor of the mandible and a part of the labium muscle. **C**. Lateral view with maxilla and labrum musculature. *lbr* labrum, ls lacinia stylet, *mab* maxilla base, *mba* mandible base, *mbl* mandible blade, *mcl* m. clypeo-labrailis, *mpa* maxillary palp, *mps* m. postgeno-stipitalis, *mpmd* m. postgeno-mandibularis dexter, *mpms* m. postgeno-mandibularis sinister, *mtc* m. tentorio-cardinalis, *mtl* m. tentorio-labialis, *mtla* m. tentorio-lacinialis, *mtm* m. tentorio-mandibularis, *mts* m. tentorio-stipitalis, *prm* prementum, *ten* tentorium.

Mouthpart muscles and their hypothesised functions are summarised in Table 2. The unpaired musculus clypeo-labralis (mcl) extended from the epistomal sulcus behind the clypeus to the labral base (Fig. 4A, C).

**Table 2:** *Philoliche rostrata*: mouthpart musculature.

Muscle	Abbreviation	Function
M. clypeo-labralis	mcl	Retractor of the labrum
M. postgeno-mandibularis d.	mpmd	Abductor of the mandible
M. postgeno-mandibularis s.	mpms	Outer adductor of the mandible
M. tentorio-mandibularis	mtm	Tentorial adductor of the mandible
M. tentorio-cardinalis	mtc	Promotor of the cardo
M. tentorio-stipitalis	mts	Tentorial retractor of the lacinia
M. tentorio-lacinialis	mtla	Tentorial retractor of the lacinia
M. postgeno-stipitalis	mps	Outer retractor of the lacinia
M. tentorio-labialis	mtl	Retractor of the labium

The paired mandibles were only present in females, and consisted of a heavy sclerotised base, bearing the sclerotised, ribbed mandible blades with a saw tooth-shaped and serrated tip (Fig. 1B, C). The mandible base additionally possessed a lateral process providing articulation with the head and a weaker sclerotised, bifurcated inner process. The overlapping mandible blades were slightly shorter than the labrum and lay beneath the labrum-epipharynx complex, closing the proximal food canal ventrally (Fig. 3A).

Three small paired conical muscles were attached to the mandible base via elongated tendons. Both the musculus postgeno-mandibularis dexter (mpmd) and the musculus postgeno-mandibularis sinister (mpms) originated at the back of the head. While the mpmd inserted on the inner process, the mpms was attached to the lateral process. The third muscle, m. tentorio-mandibularis (mtm), originated on the anterior tentorial arm and also extended to the inner process (Fig. 4A, B).

The paired maxillae each consisted of a curved and heavy sclerotised, fused cardo and stipes in the proximal proboscis region (Fig. 1A). The stipes supported the two-segmented maxillary palpus and the elongated stylet, which extended into the proximal region of the proboscis (Fig. 1, Fig. 3A). The weaker sclerotised stylet resided left and right of the hypopharynx just below the mandible blades and reached the same length. They contained a ventral and lateral sclerotised ledge which traversed the stylets over the whole length (Fig. 3A). The last third of each stylet was equipped with a toothed lateral and setaceous inner edge ending in a strongly toothed tip (Fig. 1D).

Three paired muscles were attached to the basal sclerites of the maxilla. The musculus postgeno-stipitalis (mps) extended from the back of the head to the stipes region. The massive m. tentorio-cardinalis (mtc) reached between the anterior tentorial arm and the maxillary base. Two additional muscle fibres also shared a common origin on the tentorium and branch off to their different insertions on the maxilla. The musculus tentorio stipitalis (mts) also extended to the lacinia base while m. tentorio-lacinialis (mtla) was attached to the lacinia stylet (Fig. 3A, C).

The sclerotised, lance-shaped hypopharynx was flat in cross section and traversed by the salivary duct. Connected to the posterior wall of the cibarium, it was positioned between the laciniae and below the mandible blades and reached to the tip of the labrum (Fig. 1, Fig. 3A).

#### 3.1.2 Distal nectar uptake part

The heavy sclerotised, lance shaped labium was divided into four consecutive sections (Fig. 1A): (1) the short basal membrane articulating with the head; (2) the flattened proximal part sheathing the piercing structures and dorsally covered by the labrum-epipharynx complex; (3) the elongated, distal prementum that reached over two third of the proboscis length and formed the distal food tube; and (4) the apical, paired labella.

In cross section, the prementum was traversed by nerves and small tracheae along both sides that reached also into the distal part of the proboscis (Fig. 3A). Distally, the labial food canal was formed by a dorsal tongue and groove joint of the interlocking lateral walls of the prementum, and lined with the sclerotised paraphyses and a ventral median sclerit, lengthwise separated by two grooves (Fig. 3B). Apically, the food canal discharged into the paired collecting canals of the labella, which in turn were connected to the pseudotracheal system. These micro canals on the insides of the labella were the only opening of the otherwise sealed food canal (Fig. 3C). The labella were articulated with the kappa, a paired lateral sclerit between two unsclerotized regions on the apical part of the prementum (Fig. 1E, F).

One pair of prominent hook-shaped muscles extended from inside the tentorium to the labium. The musculus tentorio-labialis originated (mtl) within a dorsolateral extension of the tentorium and inserted laterally on the labial base (Fig. 4A, B).

### 3.2 Anatomy of the suction pump

Suction pump muscles and their hypothesised functions are summarised in Table 3. The suction pump of *Philoliche* was composed of two consecutive parts with an admission valve at the proximal end of the food canal. The cibarial cavity was located in an oblique position behind the clypeus and was enclosed by a strongly sclerotised ventral and a membranous dorsal wall. The cone shaped pharynx was separated from the cibarium by a constriction and was positioned behind the frons. Both pumps were provided with a set of paired dilator muscles and the pharynx was additionally equipped with two visceral compressor muscles. The lumina of these consecutive pumping organs formed a right angle with the oesophagus, which proceeded from the posterior pharynx through the brain to the occipital foramen at the back of the head.

**Table 3:** *Philoliche rostrata*: Suction pump musculature.

Muscle	Abbreviation	Function
M. labro-epipharyngealis	mle	Epipharyngeal compressor
M. clypeo-cibarialis	mcc	Cibarial dilator
M. tentorio-pharyngealis	mtp	Pharyngeal dilator
M. fronto-pharyngealis	mfp	Pharyngeal dilator
M. pharyngealis anterior	mpa	Anterior pharynx compressor
M. pharyngealis posterior	mpp	Posterior pharynx compressor
M. postoccipitalis-oesopharyngealis	mpo	Dorsal dilator of the oesopharynx

#### 3.2.1 Cibarial pump

**Fig. 5:**
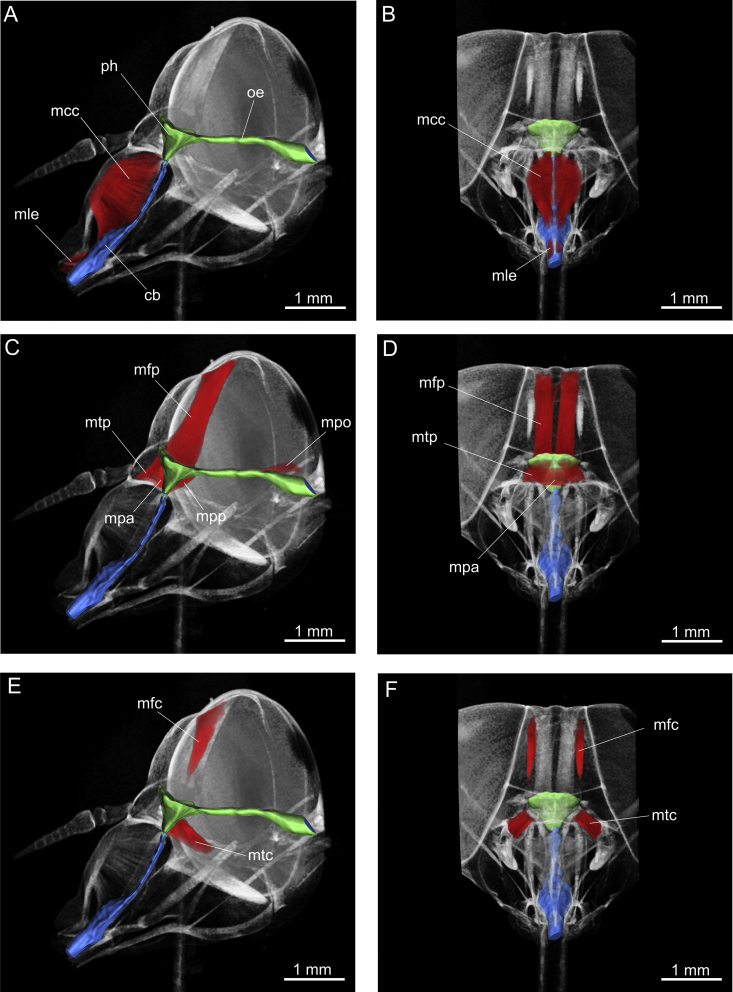
Micro-CT scans of the head of *Philoliche rostrata* displaying the cibarial pump (blue) and the pharyngeal pump with the oesophagus (green). **A**–**B**. Cibarial pump, lateral and frontal view displaying epipharyngeal compressor and cibarial dilator. **C**–**D**. Pharyngeal pump, lateral and frontal view displaying main and secondary pharyngeal dilators, pharyngeal compressors and oesopharyngeal dilator. **E**–**F**. Lateral and frontal view of cibarial protractor and retractor attached to a dorsolateral cuticular ledge on the cibarium. Cb, cibarium; mcc, musculus clypeo-cibarialis; mtp, m. clypeo-pharyngealis; mfc, musculus fronto-cibarialis; mfp, m. fronto-pharyngealis; mtc, m. tentorio-cibarialis; mle, m. labro-epipharyngealis; mpo, m. postoccipitalis-oesopharyngealis; oe oesopharynx; ph, pharynx.

The short, paired musculus labro-epipharyngealis (mle) at the base of the proboscis controlled the cibarial valve. In addition, m. clypeo-cibarialis (mcc) originated on the clypeus and inserted on the anterior cibarial wall. This massive paired muscle was responsible for operating the cibarial pump (Fig. 5A, B).

In addition, two paired muscles were attached to the sclerotised lateral wall of the cibarium, suspending it inside the head capsule. Originating laterally on the frons, m. fronto-cibarialis (mfc) attached to a dorsal tendon protracting from the cibarium ledge, lateral to the pharyngeal pump. Furthermore, the musculus tentorio-cibarialis (mtc) originated on the tentorial arm and extended obliquely to its insertion on the cibarium (Fig. 5E, F). These cibarial retractor and protractor muscles hold the pump stationary by working antagonistically against the massive dilator muscles.

#### 3.2.2 Pharyngeal pump

The pharyngeal pump was operated by two paired dilator and two unpaired compressor muscles (Fig. 5C, D). The main pharyngeal dilator, m. fronto-pharyngealis (mfp), originated on the frons and was attached to the concave dorsal wall of the pharynx. The secondary pharynx dilator, m. tentorio-pharyngealis (mtp), extended between the clypeo-frontal ridge of the tentorium and the pharynx. Two compressor muscles enclosed the anterior and posterior pharynx wall, respectively. Anteriorly, m. pharyngealis anterior (mpa) extended as a broad band between the insertion of m. clypeo-pharyngealis and the cibarial-pharyngeal constriction. The triangular m. pharyngealis posterior (mpp) extended between the oesophagus outlet and the cibarial-pharyngeal constriction on the posterior pharynx surface. In addition, a small paired muscle, m. postoccipitalis-oesophagialis (mpo), resided between the postoccipital ridge and the oesophagus near the occipital foramen (Fig. 5C, D).

### 3.3 Allometric relationships of the suction pump

**Fig. 6:**
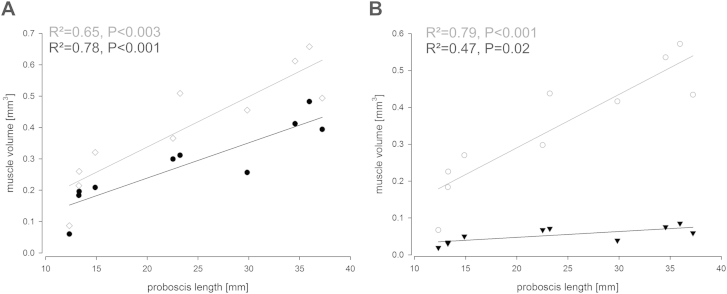
Linear regression of suction pump muscles and proboscis length in *Philoliche rostrata*. Both cibarial and pharyngeal pump muscles are positively correlated to proboscis length. **A**. Cibarial pump; cibarial dilator muscle (black circles), cibarial suspension muscles (grey diamonds). **B**. Pharyngeal pump; pharyngeal dilator muscles (grey circles), pharyngeal compressor muscles (black triangles). The compressor muscles displayed a rather weak relationship with proboscis length.

In *P. rostrata*, both cibarial and pharyngeal pump muscles displayed a significant positive relationship with proboscis length. Regarding the cibarial pump, the main dilator and both suspension muscles showed a significant positive relationship with increasing proboscis length (Fig. 6A). Furthermore, volumes of the pharyngeal dilator and compressor muscles significantly increased with proboscis length (Fig. 6B).

## 4 Discussion

### 4.1 Adaptations for feeding through an elongated proboscis

Elongated proboscides have been described in several families of flower-visiting Diptera and have evolved independently of phylogenetic relationships (Mitter, 1918, Schremmer, 1961, Dierl, 1968, Nagatomi and Soroida, 1985, Gilbert and Jervis, 1998). Usually all parts of the proboscis are equally elongated, e.g. in mosquitos (Snodgrass, 1944, Lee et al., 2009), but in nemestrinid *Prosoeca* and tabanid *Philoliche*, however, only the prementum of the labium has become extraordinary long. This represents a specialised adaptation for extracting nectar from deep floral tubes with concealed nectar sources. Furthermore, the similar morphological composition of the mouthparts in both families highlights the convergence in mouthpart adaptations associated with nectar feeding from long-tubed flowers using an extremely long proboscis. This is especially evident in the distal section of the food canal, which is formed by a single component, making this proboscid structure special amongst insects.

The conspicuous intraspecific proboscis length variations in long-proboscid flies are regarded as adaptations according to the geographic mosaic theory of coevolution (Thompson, 1994, Thompson, 2005). Selective pressure of various corolla tube lengths appear to have led to reciprocal adaptations of variations in proboscis lengths in nemestrinid flies (Anderson and Johnson, 2008, Pauw et al., 2009). Since tabanid species also belong to the same pollination system (Goldblatt and Manning, 2000b), the high variation in the elongated nectar up-taking part of the proboscis is regarded as an adaption towards different plant species within various families displaying different tube lengths that are present across the habitat (Forest et al., 2014).

Both contrasting models for the evolution of exceptionally long nectar spurs in plants, i.e. Darwin's coevolutionary race and the pollinator shift hypothesis may explain the evolution of exceptionally long-spurred flowers (Goldblatt and Manning, 2006, Whittall and Hodges, 2007). However, these models do not completely explain the evolution of exceptionally long proboscides in some flower-visiting insects. Regarded from the pollinators perspective, the diffuse co-evolution model (Johnson and Steiner, 1997, Johnson and Anderson, 2010) might be a more realistic scenario when applied to the studies that have investigated the morphological adaptation in long-proboscid flies (Karolyi et al., 2013). Similarly shaped flowers interact with certain representatives of Nemestrinidae and Tabanidae with similar proboscis lengths. The convergent evolution of exceptionally long proboscides in these insects might be triggered by the dominant community of plants in a habitat. Assuming that these flies are unable to switch to other nectar sources, the spur lengths of available flowers represent the natural selective force that is responsible for the convergent evolution of exceptionally long-proboscid flies. The second precondition is that the involved insects possess the potential of elongating a rather simple structure of the mouthparts in a way that benefits of an elongated proboscis balance the anatomical costs of obtaining such lengths. This is seen in nemestrinid flies and now here in tabanid flies where only the distal parts of the proboscis - that is formed by a single unit - are adapted to the nectar spur length. However, the positive scaling relationships between proboscid length and suction pump muscle size, as seen in both long-proboscid Nemestrinidae (Karolyi et al., 2013) and Tabanidae, indicates that obtaining an elongated proboscid requires some cost through reciprocal anatomical modifications. Karolyi et al. (2013) showed the behavioural cost related to increased feeding time with an elongated proboscis; however, how having an elongated proboscis effects overall fly morphology (muscle mass, wing length, etc.) and body size, and ultimately the evolution and speciation of long-proboscid flies is unknown.

### 4.2 Blood-feeding and nectar-sucking

Both males and females from the genus *Philoliche* use their elongated mouthparts to draw nectar from long-spurred flowers. In contrast, blood feeding females only use the relatively short piercing mouthparts (Goodier, 1962, Morita, 2007). Depending on the feeding habit, two different proboscis positions can be distinguished. While feeding from flowers, the labium is inserted straight forward into the corolla or in a slightly angled downward position (Johnson and Morita, 2006). During blood feeding however, the labium is flapped backwards oblique to the main body axis and only the mandibles and maxillary structures are used to pierce the skin and suck up blood through the epipharyngeal food canal (Tetley, 1917, Mitter, 1918, Goodier, 1962, Dierl, 1968).

In order to maintain a pressure drop in the extraordinarily long food canal, both the proximal and distal food canal require adequate sealing structures (Karolyi et al., 2012). In *Philoliche*, the labrum forms the dorsal cover of the piercing structures and contains the epipharyngeal food canal. Similar to the labral retractor of short-proboscid Tabanidae (Bonhag, 1951), labral muscles insert within the labral base via tendon-like fibres in *Philoliche*. Contracting this muscle possibly presses the labrum against the mandible blades and the hypopharynx, sealing the food canal dorsally. The unpaired clypeolabral muscle could be regarded as an ancestral character state for Diptera (Schneeberg and Beutel, 2011). In addition, the proximal food canal is ventrally sealed by the overlapping mandible blades, while the distal food canal is sealed by the interlocking lateral walls of the prementum, forming a dorsal tongue and groove joint. This results in a concealed, straw-like nectar extracting apparatus, which leads to the pseudotracheal system of the labella. There, nectar is taken up and then transported through the elongated premental food canal into the head via the main sucking pump (Karolyi et al., 2012). As in *Prosoeca*, the labella of *Philoliche* were remarkably short, heavily sclerotised and sclerites required for movement, as well as the labella musculature were reduced. However, limited movability of the labella might represent a particular adaptation for nectar uptake from narrow, elongated flower tubes. Such flower traits prevent the labella from spreading apart, and nectar intake is therefore performed with closed labella (Karolyi et al., 2012).

During blood-feeding, the initial laceration of the host skin is achieved by scissor like movement of the apical serrated mandible blades (Bonhag, 1951, Chaudonneret, 1990). The mandible abductor pulls the mandible blades slightly outwards, while contractions of the adductor muscles move the blades back into the initial position. Similar to short-proboscid Tabanidae, the maxilla stylets of *Philoliche* are also responsible for the initial puncturing of the skin (Bonhag, 1951), representing the piercing structures that are actively inserted into the host skin (Matsuda, 1965, Krenn and Aspöck, 2012). Protraction and retraction of the stylets are most likely accomplished by bending and stretching the curved maxillary base due to the retractor muscles of the laciniae and their antagonists, the massive promotors of the cardo (Bonhag, 1951). Continuing in this way, the stylets work like a push drill that penetrates the host skin with the toothed tip region, and the sclerotised, setaceous inner edge possibly widens the puncture wound. In addition, the hypopharynx forms the floor of the epipharyngeal food canal and acts similarly to a syringe that injects saliva into the wound. Overall, it can be concluded that the function of the piercing structures of short-tonged Tabanidae are similar to that of long-proboscid horse flies.

### 4.3 Suction pump design

The feeding apparatus of *Philoliche* combines the two level fluid system of a straw-like proboscis (Monaenkova et al., 2012) with the systaltic motion of a two-part suction pump. Within this system, capillary action of the nanosponge-like labella and a pump operated drinking straw are responsible for filling the food canal. *Philoliche*, like Nemestrinidae and Lepidoptera, most likely relies on a pressure gradient along the elongated proboscis (Karolyi et al., 2012, Monaenkova et al., 2012).

The suction pump of *Philoliche* combines two pumping organs regulated by a musclulated cibarial valve and a constriction between the cibarium and the pharynx; dividing the sucking process into three functional phases. During the first phase, the functional mouth opens at the cibarial valve and the cibarium is expended by contractions of the cibarial dilator that pulls the elastic anterior wall towards the clypeus. Due to the expanding cibarial chamber, fluid is sucked out of the food canal into the cibarium. In the second phase, relaxation of the cibarial pump and combined contraction of the pharyngeal dilators draw nectar through the constriction valve at the true mouth into the pharyngeal chamber. During the last phase, the pharyngeal dilator muscles relieve tension and the compressor muscles close the true mouth and push the nectar into the oesophagus. Finally, dorsal dilators open the distal oesophagus valve to the midgut.

### 4.4 Evolution of nectar feeding

Both Tabanidae and Nemestrinidae belong to the lower brachyceran linages (Wiegmann et al., 2011), possibly reflecting the starting point for the evolution of brachyceran mouthparts. Although the radiation of these groups coincides with the origin of flowering plants (Smith et al., 2010), piercing mouthparts are regarded as plesiomorphic in Diptera (Downes, 1971). However, a basic duality in diet has been described before in piercing flies (Downes, 1958) and consequently the female mouthparts have a dual role of puncturing integument for blood-feeding and taking up nectar from flowers (Labandeira, 2010).

Female long-proboscid *Philoliche* flies have to deal with both nectar and blood. The dual task is accomplished by only a few adaptations to blood feeding, such as the mechanism to separately bend the labium backwards to expose the piercing structures. This mechanism has not been found in short tongued Tabanidae.

The piercing mouthparts of long-proboscid *Philoliche* can be characterised as follows: blade-like mandibles, the hypopharynx, lacinia stylets and a lance-shaped labrum that are about equal in length. Overall, the mouthparts of Tabanidae are similar to those of biting Nematocera and feature a characteristic piercing apparatus comprising the piercing structures (Jobling, 1976, Nagatomi and Soroida, 1985). Tabanidae are considered to reflect the starting point for the evolution of brachyceran mouthparts (Labandeira, 1997, Karolyi et al., 2012). Compared to Nematocera, their feeding apparatus differs especially in the conspicuously developed labella and the position of the pharyngeal pump, which is operated by precerebral muscles (Downes, 1958).

In contrast, the proboscis of Cyclorrhapha is usually divided into three parts: rostrum, haustellum and labella (Matsuda, 1965). Furthermore, mandibles are usually absent in cyclorrhaphous Diptera as well as in many families of basal Brachycera. Well-developed, extant mandibles occur only within the Tabanomorpha, including female Tabanidae and the blood-sucking *Spaniopsis* (Rhagionidae) (Nagatomi and Soroida, 1985).

Proboscis length and shape, as well as the morphology of the suction pump of long-proboscid *Philoliche* appear outwardly comparable with strictly nectar-feeding *Prosoeca* (Morita, 2011, Karolyi et al., 2012). Compared to long-proboscid *Prosoeca*, however, our detailed study has revealed noticeable differences in muscle number and size. The paired muscle controlling the cibarial-pharyngeal valve is missing in *Philoliche* and the pharyngeal compressors are noticeable smaller. These muscles have been regarded as special adaptations to nectar feeding (Karolyi et al., 2013).

Kunte (2007) suggested that strictly nectar-feeding insects should have a greater cibarial muscle mass to increase the rate of nectar uptake. These assumptions have already been supported for nectar-feeding Nemestrinids (Karolyi et al., 2013) and butterflies (Kunte, 2007). The positive linear relationship between proboscis length and suction pump dimensions in *Philoliche* was expected as the two species considered here are highly active fliers reliant on nectar sources for energy intake. In addition, these positive correlations underline the importance of both pumps in tabanid flies and indicate that a longer proboscis demands larger pumping organs. In comparison to the massive dilator muscles of the suction pump, the volume of the pharyngeal compressor muscles are very small. Therefore, the weak relationship between proboscis length and compressor muscles suggest that an enlargement of the main dilator muscles with increasing proboscis length is more important compared to enlarged compressors.

As in long-proboscid Nemestrinidae, the elongated prementum of *Philoliche* is thought to have evolved by “diffuse coevolution” (Janzen, 1980) along with long-tubed flower guilds (Pauw et al., 2009, Anderson and Johnson, 2009), while the piercing structures retain a length that is adapted to host skin. Therefore, the evolution of a distal nectar up-taking proboscis in long-proboscid *Philoliche* is regarded as an adaptation that gave exclusive access to nectar-rewarding long-tubed flowers, while the proximal piercing part remained unmodified in comparison to other Tabanidae (Bonhag, 1951).

This study has shown the required structural adaptation of an elongated proboscis and the associated suction pump, including considerations about the duality of blood- and nectar feeding. Together with recent studies about the feeding apparatus of long-proboscid Nemestrinidae (Karolyi et al., 2012, Karolyi et al., 2013) and Lepidoptera (Bauder et al., 2011, Bauder et al., 2013), results presented here contribute to the understanding of the functional morphology and evolution of extremely long mouthparts in flower-visiting insects and underlie the morphological convergence in mouthpart structure in different lineages of long-proboscid flies adapted to feeding from long-tubed flowers.

## References

[bib1] Anderson B., Johnson S.D. (2008). The geographical mosaic of coevolution in a plant-pollinator mutualism. Evolution.

[bib2] Anderson B., Johnson S.D. (2009). Geographical covariation and local convergence of flower depth in a guild of fly-pollinated plants. New Phytol..

[bib3] Bauder J.A.-S., Handschuh S., Metscher B.D., Krenn H.W. (2013). Functional morphology of the feeding apparatus and evolution of proboscis length in metalmark butterflies (Lepidoptera: Riodinidae). Biol. J. Linn. Soc..

[bib4] Bauder J.A.S., Lieskonig N.R., Krenn H.W. (2011). The extremely long-tongued Neotropical butterfly *Eurybia lycisca* (Riodinidae): proboscis morphology and flower handling. Arthropod Struct. Dev..

[bib5] Bock C. (1987).

[bib6] Bonhag P.F. (1951). The skeleto-muscular mechanism of the head and abdomen of the adult horsefly (Diptera: Tabanidae). Trans. Am. Entomol. Soc..

[bib7] Borrell B.J., Krenn H.W., Herrel A., Speck T., Rowe N.P. (2006). Ecology and Biomechanics: a Mechanical Approach to the Ecology of Animals and Plants.

[bib8] Chaudonneret J. (1990).

[bib9] Davis N.T., Hildebrand J.G. (2006). Neuroanatomy of the sucking pump of the moth, *Manduca sexta* (Sphingidae, Lepidoptera). Arthropod Struct. Dev..

[bib10] Dierl W. (1968).

[bib11] Downes J.A. (1958). The feeding habits of biting flies and their significance in classification. Annu. Rev. Entomol..

[bib12] Downes J.A., Fallis A.M. (1971). Ecology and Physiology of Parasites.

[bib13] Eberhard S.H., Krenn H.W. (2005). Anatomy of the oral valve in nymphalid butterflies and a functional model for fluid uptake in Lepidoptera. Zool. Anz. J. Comp. Zool..

[bib14] Forest F., Goldblatt P., Manning J.C., Baker D., Colville J.F., Devey D.S., Jose S., Kaye M., Buerki S. (2014). Pollinator shifts as triggers of speciation in painted petal irises (Lapeirousia: Iridaceae). Ann. Bot..

[bib15] Gilbert F., Jervis M. (1998). Functional, evolutionary and ecological aspects of feeding-related mouthpart specializations in parasitoid flies. Biol. J. Linn. Soc..

[bib16] Goldblatt P., Manning J.C. (2000).

[bib17] Goldblatt P., Manning J.C. (2000). The long proboscid fly pollination system in southern Africa. Ann. Mo. Bot. Gard..

[bib18] Goldblatt P., Manning J.C. (2006). Radiation of pollination systems in the Iridaceae of sub-Saharan Africa. Ann. Bot..

[bib19] Goldblatt P., Manning J.C., Bernhardt P. (1995). Pollination biology of *Lapeirousia* subgenus *Lapeirousia* (Iridaceae) in southern Africa; floral divergence and adaptation for long-tongued fly-pollination. Ann. Mo. Bot. Gard..

[bib20] Goodier R. (1962). Blood feeding by *Philoliche (Dorcaloemus) silverlocki* Austen (Diptera, Tabanidae). Nature.

[bib21] Harrell F. (2013).

[bib22] Janzen D.H. (1980). When is it coevolution. Evolution.

[bib23] Jobling B. (1976). On the fascicle of blood-sucking Diptera in addition a description of the maxillary glands in *Phlebotomus papatasi*, together with the musculature of the labium and pulsatory organ of both the latter species and also of some other Diptera. J. Nat. Hist..

[bib24] Johnson S., Anderson B. (2010). Coevolution between food-rewarding flowers and their pollinators. Evol. Educ. Outreach.

[bib25] Johnson S.D. (2010). The pollination niche and its role in the diversification and maintenance of the southern African flora. Philos. Trans. R. Soc. B Biol. Sci..

[bib26] Johnson S.D., Johnson K. (1993). Beauty and beast: a Cape orchid pollinated by horseflies. Veld Flora.

[bib27] Johnson S.D., Morita S. (2006). Lying to Pinocchio: floral deception in an orchid pollinated by long-proboscid flies. Bot. J. Linn. Soc..

[bib28] Johnson S.D., Steiner K.E. (1997). Long-tongued fly pollination and evolution of floral spur length in the *Disa draconis* complex (Orchidaceae). Evolution.

[bib29] Karolyi F., Morawetz L., Colville J.F., Handschuh S., Metscher B., Krenn H.W. (2013). Time management and nectar flow: flower handling and suction feeding in long-proboscid flies (Nemestrinidae: Prosoeca). Naturwissenschaften.

[bib30] Karolyi F., Szucsich N.U., Colville J.F., Krenn H.W. (2012). Adaptations for nectar-feeding in the mouthparts of long-proboscid flies (Nemestrinidae: Prosoeca). Biol. J. Linn. Soc..

[bib31] Kim B.H., Kim H.K., Lee S.J. (2011). Experimental analysis of the blood-sucking mechanism of female mosquitoes. J. Exp. Biol..

[bib32] Kim W., Gilet T., Bush J.W.M. (2011). Optimal concentrations in nectar feeding. Proc. Natl. Acad. Sci..

[bib33] Kingsolver J.G., Daniel T.L. (1979). On the mechanics and energetics of nectar feeding in butterflies. J. Theor. Biol..

[bib34] Krenn H.W., Aspöck H. (2012). Form, function and evolution of the mouthparts of blood-feeding Arthropoda. Arthropod Struct. Dev..

[bib35] Krenn H.W., Plant J.D., Szucsich N.U. (2005). Mouthparts of flower-visiting insects. Arthropod Struct. Dev..

[bib36] Kunte K. (2007). Allometry and functional constraints on proboscis lengths in butterflies. Funct. Ecol..

[bib37] Labandeira C.C. (1997). INSECT mouthparts: ascertaining the paleobiology of insect feeding strategies. Annu. Rev. Ecol. Syst..

[bib38] Labandeira C.C. (1998). How old is the flower and the fly?. Science.

[bib39] Labandeira C.C. (2010). The pollination of mid Mesozoic seed plants and the early history of long-proboscid insects. Ann. Mo. Bot. Gard..

[bib40] Lee S.J., Kim B.H., Lee J.Y. (2009). Experimental study on the fluid mechanics of blood sucking in the proboscis of a female mosquito. J. Biomech..

[bib41] Lehane M.J. (2005). The Biology of Blood-sucking in Insects.

[bib42] Matsuda R. (1965).

[bib43] Metscher B. (2009). MicroCT for comparative morphology: simple staining methods allow high-contrast 3D imaging of diverse non-mineralized animal tissues. BMC Physiol..

[bib44] Mitter J.L. (1918). Note on the method of feeding of *Corizoneura* (*Pangonia*) *Longirostris* Hardwick, with a description of the mouth parts. Indian J. Med. Res..

[bib45] Monaenkova D., Lehnert M.S., Andrukh T., Beard C.E., Rubin B., Tokarev A., Lee W.-K., Adler P.H., Kornev K.G. (2012). Butterfly proboscis: combining a drinking straw with a nanosponge facilitated diversification of feeding habits. J. R. Soc. Interface.

[bib46] Morita S. (2007).

[bib47] Morita S. (2008). A revision of the *Philoliche aethiopica* species complex (Diptera: Tabanidae). Afr. Invertebr..

[bib48] Morita S.I. (2011). Repeatability and precision in proboscis length measurements for long proboscid flies. Zootaxa.

[bib49] Nagatomi A., Soroida K. (1985). The structure of the mouthparts of the orthorrhaphous Brachycera (Diptera) with special reference to blood-sucking. Beitr. Entomol..

[bib50] Pauw A., Stofberg J., Waterman R.J. (2009). Flies and flowers in Darwin's race. Evolution.

[bib51] R Core Team (2012).

[bib52] Ren D. (1998). Flower-associated brachycera flies as fossil evidence for jurassic angiosperm origins. Science.

[bib53] Schneeberg K., Beutel R.G. (2011). The adult head structures of Tipulomorpha (Diptera, Insecta) and their phylogenetic implications. Acta Zool..

[bib54] Schremmer F. (1961).

[bib62] Smith S.A., Beaulieu J.M., Donoghue M.J. (2010). An uncorrelated relaxed-clock analysis suggests an earlier origin for flowering plants. Proc. Natl. Acad. Sci. U. S. A..

[bib55] Snodgrass R.E. (1944). The feeding apperatus of biting and sucking insects affecting man and animals. Smithson. Misc. Collect..

[bib56] Tetley H. (1917). The structure of the mouth-parts of *Pangonia longirostris* in relation to the probable feeding-habits of the species. Bull. Entomol. Res..

[bib57] Thompson J.N. (1994).

[bib58] Thompson J.N. (2005).

[bib59] Wheeler B. (2010).

[bib60] Whittall J.B., Hodges S.A. (2007). Pollinator shifts drive increasingly long nectar spurs in columbine flowers. Nature.

[bib61] Wiegmann B.M., Trautwein M.D., Winkler I.S., Barr N.B., Kim J.-W., Lambkin C., Bertone M.A., Cassel B.K., Bayless K.M., Heimberg A.M., Wheeler B.M., Peterson K.J., Pape T., Sinclair B.J., Skevington J.H., Blagoderov V., Caravas J., Kutty S.N., Schmidt-Ott U., Kampmeier G.E., Thompson F.C., Grimaldi D.A., Beckenbach A.T., Courtney G.W., Friedrich M., Meier R., Yeates D.K. (2011). Episodic radiations in the fly tree of life. Proc. Natl. Acad. Sci..

