# Corrigendum to “One proboscis, two tasks: Adaptations to blood-feeding and nectar-extracting in long-proboscid horse flies (Tabanidae, Philoliche)” [Arthropod Struct. Dev. 43 (2014) 403–413]

**DOI:** 10.1016/j.asd.2014.10.002

**Published:** 2015-01

**Authors:** Florian Karolyi, Jonathan F. Colville, Stephan Handschuh, Brian D. Metscher, Harald W. Krenn

**Affiliations:** a Department of Integrative Zoology, University of Vienna, Faculty of Life Science, Althanstrasse 14, 1090 Vienna, Austria; b South African National Biodiversity Institute, Kirstenbosch Research Centre, Private Bag X7, Claremont, Cape Town, South Africa; c VetCore Facility for Research, University of Veterinary Medicine, Vienna, Austria; d Department of Theoretical Biology, University of Vienna, Faculty of Life Science, Althanstrasse 14, 1090 Vienna, Austria

The authors regret that the following project number stated in the Acknowledgments section is incorrect. The correct number should have been P 222 48-B17. The corrected text appears below.

The authors would like to apologize for any inconvenience this may have caused to the readers of the journal.

